# Ash analyses of bio-coal briquettes produced using blended binder

**DOI:** 10.1038/s41598-020-79510-9

**Published:** 2021-01-12

**Authors:** A. A. Adeleke, J. K. Odusote, P. P. Ikubanni, T. A. Orhadahwe, O. A. Lasode, A. Ammasi, K. Kumar

**Affiliations:** 1 Department of Mechanical Engineering, Landmark University, Omu Aran, Nigeria; 2 Department of Materials and Metallurgical Engineering, University of Ilorin, Ilorin, Nigeria; 3 Department of Mechanical Engineering, University of Ibadan, Ibadan, Nigeria; 4 Department of Mechanical Engineering, University of Ilorin, Ilorin, Nigeria; 5 Metal Extraction and Recycling Division, National Metallurgical Laboratory, Jamshedpur, India

**Keywords:** Engineering, Mechanical engineering, Materials for energy and catalysis

## Abstract

The behaviour of ash of fuel affects its thermal efficiency when in use. The ash analyses of bio-coal briquettes developed from lean grade coal and torrefied woody biomass have received limited intensive study. Therefore, the present study aims at analysing the ashes of briquette made from lean grade coal and torrefied woody biomass using blended coal tar pitch and molasses as the binder. Bio-coal briquettes were produced from coal and torrefied biomass in various hybrid ratios. Ashing of various briquettes was done in a muffle furnace at 850 °C for 3 h. Mineral phases of the ash were identified using an X-ray Diffractometer (XRD), while the mineral oxides were obtained using an X-ray Fluorescence Spectrometer. The AFT700 Furnace was used with its AFT700 software to evaluate the ash fusion temperatures of the ashes. The XRD patterns look similar, and quartz was found to be the dominant mineral phase present in the raw coal and bio-coal briquettes. The SiO_2_ (57–58%), Al_2_O_3_ (19–21%), and Fe_2_O_3_ (8–9%) were the major oxides observed in the ashes. The final fusion temperatures of the ashes range from 1300–1350 °C. The compositions of the ashes of the bio-coal briquettes are classified as detrital minerals. It was concluded that the addition of torrefied biomass (≤ $10\%)$and blended binder ($\le$ 15%) to coal gave a negligible impact on the ashes of the resultant bio-coal briquettes.

## Introduction

The impact of man’s activity on nature has led to a change in the climatic condition of the world, which is evident by the presence of widespread natural disasters in different parts of the world. The continuous quest by man for energy to drive industrial activities led to the use of forest resources to generate energy^1,2^. Thus, the increase in desert encroachment and the extinction of wildlife species. Perennial flooding, earthquakes, tsunamis, and wildfires are some of the natural havocs man has faced as a result of these activities^3^. Meanwhile, the use of fossil fuel, as a source of energy, is adjudged as one of the primary causes of the thinning away of the ozone layer due to the release of harmful CO_x_ and NO_x_ gases, thereby leading to global warming^4–6^. This is one major rationale that has driven many researchers to consider eco-friendly alternative fuel. Researches have shown that biomass can be compacted into a potent energy source through briquetting^3,7–12^. The use of loose biomass will not only prevent the release of harmful gases into the atmosphere but will also prevent further encroachment into forest resources, while at the same time ensuring that our communities are kept clean by converting these biomasses into energy products^10^. However, these biomasses have been reported to have ash compositions that may weaken its usefulness for energy generation. Hence, the need to use biomass as a partial replacement for fossil fuel (coal) in energy generation^11,13–15^. The ash content describes the product of incomplete combustion, which may be majorly minerals but could still contain some amount of organic or other oxidizable residues^16^. The chemical composition determines the melting point of the fuel ash. Usually, the ash comprises of compounds of metals such as sodium, vanadium, magnesium, among others. The ash content and composition often lower the calorific value of a fuel^17^. High ash content is destructive in boiler applications due to the clinging of the ash components to the surfaces of the boiler, which can lead to corrosion^18^. This underscores the need to carry out thorough ash analysis of briquettes before deploying such in any application. Several researchers have carried out ash analyses using various techniques for different fuel samples. Most researchers analysed mainly for the percentage content of ash in fuel using conventional ash content test specified by ASTM D3174 standard ^3,15,19,20^. To properly elucidate the role of the ash content of fuel during the application, a more detailed analysis is required. For instance, Markiewicz-Keszycka et al.^21^ carried out ash analysis using laser-induced breakdown spectroscopy of free flours and posited that ash content is a determinant of the hygroscopicity and coloration of products. This method was also used by Zhang et al.^22^ in classifying coal ash. Qin et al.^23^ deployed optical heating stage microscopy to investigate the ash characteristics of biomass and coal. It was reported that the method could measure the dimension change of the solid particles during the ash fusion test. Shoji et al.^24^ also used a convolutional neural network and probability in classifying the ash obtained from volcanic eruption. The method was able to capture ash particles of multiple basal shapes. Xing et al.^25^ carried out biomass ash analysis using X-ray fluorescence and wet chemical analysis, and established a relationship between the two methods. These techniques helped in placing a significant understanding on the composition of biomass and coal ashes. However, there are limited studies on the ash behaviour of composite briquette produced from coal and biomass using organic binders. This is because the majority of the previous research works focused more on the physicomechanical integrity of the briquette of coal and biomass with little efforts on their ash analyses^26–32^. However, there is increasing attention on coal ash characterization and modification because of its importance during the combustion process. Li and Fang^33^ investigated on the ash fusion characteristics of high aluminium coal and its modification behaviour. It was observed that ash fusion temperature (AFTs) of mixed ashes was mainly dependent on mineral composition and their transformation. It was further stated that calcium and iron in blended coal evolve into eutectics and amorphous matter, which decreased the AFT. Li and Fang^34^ modified the ash behaviour of lignite by adding different biomass. It was concluded that increase mass ratio of biomass led to an increase in the low-melting-point mineral and their eutectics at high temperatures. Hence, a reduction in the AFTs of the mixed lignite and biomass ash. Li et al.^35^ also studied the effect of vanadium on the ash fusibility of petroleum coke. It was concluded that an increase in vanadium trioxide (V_2_O_3_) led to the formation of high melting spinel NiAl_2_O_4_ and V-bearing amorphous, which in turn increased the AFTs. Ash deposition behaviour of straw modified by adding lignite was experimentally investigated by Li et al.^36^. Husheng and Huolinle lignites were added to straw (corn stalk, wheat straw, cotton stems, and soybean stalk) in different mass ratios. It was reported that increased quartz content of the ash and high-melting-point mullite generation resulted in a decreased mass of ash deposition of the straw with an increased mass ratio of lignite. Ma et al.^37^ also attempted regulating the ash fusion characteristics of high AFT coal by adding bean straw. The bean straw was reported to effectively reduce the AFTs of Jiaozuo and Zaozhuang coals for its numerous basic components. Several researchers have worked on the characterization and modification of the AFTs of coal blended with biomass. More so, there are also a lot of works on the transformation behaviour of the various constituents in coal ash. However, little or no work has been done on ash analyses of bio-coal briquettes produced majorly from organic blended binders. The continuous interest in reducing the usage of coal as feedstock for both energy and metallurgical applications has paved way for intense investigation on biomass in all ranks^38^. Therefore, the present study aims to analyse (composition, ash mineral phases, AFTs, and oxide ratios) the ash of bio-coal briquettes produced from lean grade coal and torrefied woody biomass using blended coal tar pitch and molasses as the binder. This is to ascertain the quality of the bio-coal briquettes during its thermochemical conversion as fuel in energy and metallurgical applications.

## Materials and methods

### Materials

The raw materials used to produce bio-coal briquettes in this study were lean grade subbituminous coal fines, Melina wood dust, coal tar pitch, and molasses. The coal fines were collected from Okaba mine, Nigeria (7° 23′ 0′′ N, 7° 44′ 0′′ E), sun-dried, and screen to a particle size below 0.7 mm. The Melina wood dust was sun-dried for five days (5 h/ day) and screen to a particle size below 2 mm. It was then torrefied in a tubular furnace at 260 °C for 60 min to improve the energy density and hydrophobic property^30^. The torrefied melina dust was pulverized and screened to a particle size below 0.7 mm. The coal tar pitch was used directly at a particle size of less than 0.70 mm.

### Methods

#### Bio-coal briquette formulation and production

The bio-coal briquette production process was reported by Adeleke et al.^30^. Raw materials used were in accordance with the typical formulation shown in Table 1 for the total briquette weight. Representatively, 8P-7 M for blended binder composition implied that 8% pitch and 7% molasses were added as part of the total briquette weight, as shown in Table 1. Similarly, 90:10 is a tag for hybrid ratio where coal fine is 90% and torrefied biomass is 10% of the total weight of the base materials. The blends of coal fines, torrefied biomass, binder, and water were thoroughly mixed to obtain homogeneity. Briquetting of 25 g of the blends was carried out in a 25 mm internal diameter cylindrical steel die under a hydraulic press with a load of 28 MPa. Briquettes of various configurations and hybrid ratios were produced. Briquettes were initially cured at room temperature for 24 h and then further cured in a muffle furnace (inert condition) at 200 °C for a residence time 60 min. The briquettes were allowed to cool in a desiccator after removal from the furnace. The physicomechanical, proximate, ultimate, and calorific analyses of these bio-coal briquettes have been reported in another study^30^. Ashing of pulverized bio-coal briquettes was carried out in a muffle furnace at 815 °C for 3 h.

**Table 1:** Formulation of bio-coal briquettes using different blended binder ratios.

Hybrid ratio	Base materials		Binders (%)		Water (%)
	Coal (%)	TB (%)	Pitch	Molasses	
97:3	97	3	5	5	10
97:3	97	3	8	7	10
97:3	97	3	10	5	10
97:3	97	3	5	10	10
95:5	95	5	5	5	10
95:5	95	5	8	7	10
95:5	95	5	10	5	10
95:5	95	5	5	10	10
90:10	90	10	5	5	10
90:10	90	10	8	7	10
90:10	90	10	10	5	10
90:10	90	10	5	10	10

#### Phase identification

X-ray diffraction (XRD) method was used to identify different mineral phases present in the ashes of the bio-coal briquettes. Powdered samples were scanned at 2 $\uptheta$ from 10—90 $^\circ$ using Cu-${K}_{\propto }$ radiation filtered with Ni ($\lambda$ of 1.5426 $\AA$) at the scan rate of 0.02 s/step in an X-ray power Diffractometer (D8 Discover, Bruker, Germany). Phases present in pulverized bio-coal briquettes samples were determined by JCPDF (Joint committee powder diffraction file) software with standard XRD patterns of various elements and compounds of powder samples.

#### Ash compositions

The compositional (oxides) analysis of the bio-coal briquette ashes was done with the aid of an X-ray fluorescence *(*XRF*)* spectrometer (Bruker S8 TIGER model). Briquette ash of 8 g was thoroughly mixed with 2 g of wax (binder). Pellet of 34 mm diameter (1 mm thick) was produced from the mixture. The sample was then placed in sample holder and transferred into the XRF for analyses. Spectra plus launcher was used to collect ash compositions^31,39^.

#### Ash fusion test

Ash fusion temperature (AFT) was carried out in accordance with ASTMD 1857–04 standard^40^. The ash (1 g) was mixed with dextrin solution to be formable into cone in shape plate. The formed cones were allowed to dry for 4–5 min in the sample holder. The AFT (AF700) furnace was purged with N_2_ and O_2_ gases (50/50) at 2.2—2.5 L/min. At 400 °C, the sample was placed in the furnace while the process was monitored with an AFT700 software to evaluate the initial deformation temperature (DT), softening temperature (ST), hemispherical temperature (HT), and final fusion temperature (FT)^31,39^.

## Results and discussion

### XRD analysis

**Figure 1:**
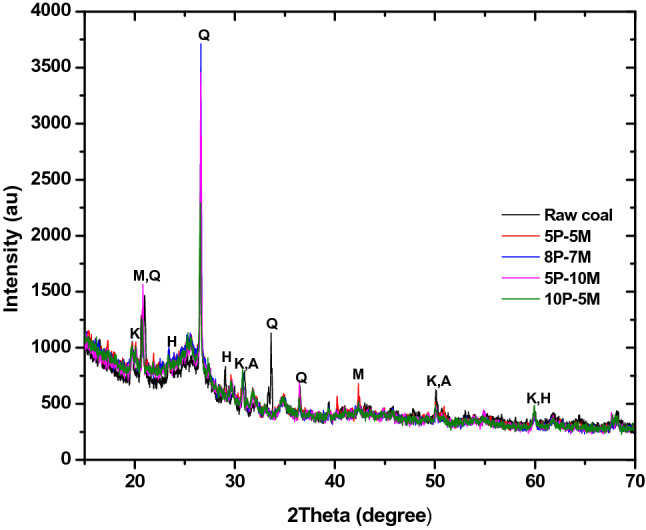
Diffractograms of bio-coal briquette produced from 97:3 hybrid ratio.

**Figure 2:**
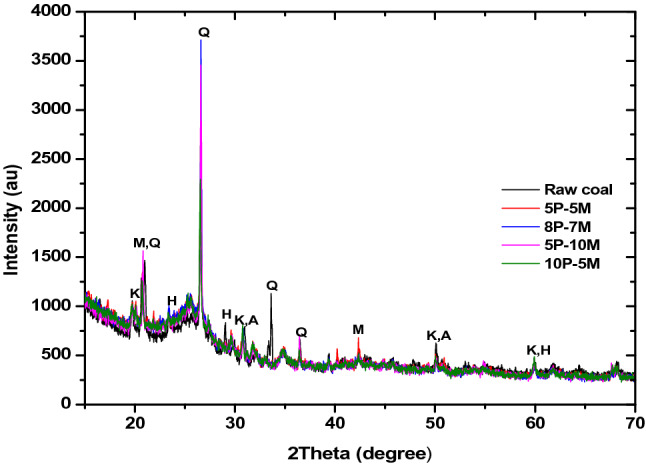
Diffractograms bio-coal briquette produced from 95:5 hybrid ratio.

**Figure 3:**
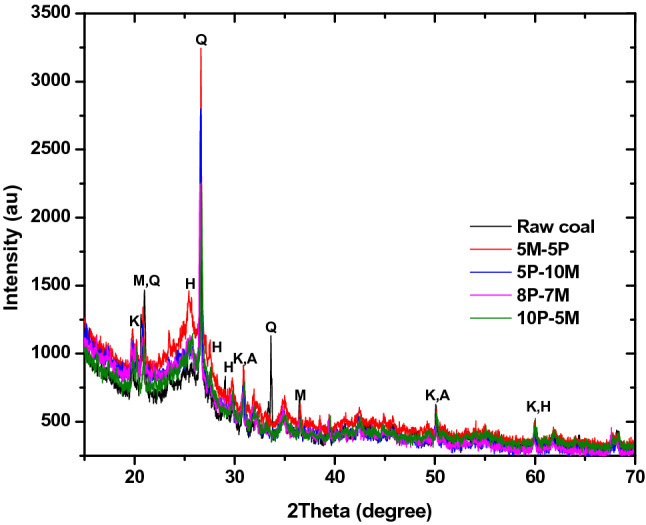
Diffractograms of bio-coal briquette produced from 90:10 hybrid ratio.

The major mineral phases present in the ashes of the raw coal and bio-coal briquettes are shown in the diffractograms (XRD pattern) in Figs. 1, 2 and 3. The XRD patterns of the ashes of the bio-coal briquette showed similar mineral phases. It mainly indicates the presence of Quartz (Q), Mullite (M) and Hematite (H), Kaolinite (K), and Anatase (A), and the lower temperature oxides of titanium (rutile) were also observed in the diffractograms. These peaks in the patterns were identified through the JCPD file and previous studies^39,41^. The XRD patterns for all the different hybridization ratios of the bio-coal briquettes were similar. Quartz (Q) was seen as the major mineral phase that is dominant in all the XRD patterns. One of the detrital minerals that enhances abrasion-erosion forms low-temperature eutectics, and decrease the combustion efficiency is the hard quartz. Others are the rutile, feldspar, and corundum. However, the quartz present in bio-coal briquettes are well balanced with other minerals that mitigate against these behaviours. The lean grade coal dictated the dominant phases present in the bio-coal briquettes. The additions of torrefied biomass and different organic binders at different ratios contributed no significant alteration in the mineral phases of the ashes of the bio-coal briquettes. This may be as a result of the low ash content of the torrefied biomass and the organic binders^30^. There was no transformation in phases of mineral present in the ashes of the bio-coal briquettes. This could be as a result of low -temperature curing used during the briquetting process. An increase in the curing temperature of the bio-coal briquettes above 400 °C could lead to mineral phase transformations where rutile, kaolinite, among others, may be oxidized to form different oxides^41^. The transformation of various oxides to other forms could pose a major setback leading to inefficient combustion process when bio-coal briquettes are in use. Identification of some other minerals by XRD only in a multi-component system such as the lean grade coal and bio-coal briquettes is difficult due to peak overlapping^42^. However, the presence of other mineral phases in form of oxides was visibly detected and quantified using XRF analysis.

### XRF analysis

The composition of the major oxides found in the raw coal and bio-coal briquette ashes are SiO_2_ (57—58%), Al_2_O_3_ (19—21%), Fe_2_O_3_ (8—9%), K_2_O, MgO, and CaO (2—3%), Na_2_O (0.2%) and TiO_2_ (1—2%), as presented in Table 2. The presence of ZnO and CuO was very small in ppm. The presence of these oxides in the coal and bio-coal briquette has been reported in previous studies^39,41–43^. The presence of these oxides in different ratios serves as indices for different coal ash behaviours such as slagging, fouling, and abrasion. These were further discussed in detail in the other subsection. The SiO_2_ and Al_2_O_3_ contents of the raw coal and bio-coal briquettes are within the acceptable range for the ash of fuel suitable for energy and metallurgical applications^39,42^. The SiO_2_ and Al_2_O_3_ variations showed that an increase in torrefied biomass within the bio-coal briquette composition had an abysmal effect on the ash. The report of Li et al.^36^ also showed that increase in the mixed ratio of Husheng lignite (HL) when added to corn stalk led to a slight increase in the Al_2_O_3_ and SiO_2_ content of the mix. The percentage of Fe_2_O_3_ slightly increased with an increase in HL within the admixture. This implied that an increase in biomass (corn stalk) decreased the percentage composition of these major oxides in the admixture. Variation in blended binder composition also has negligible effects on the disparity in the values of these oxides. The pattern in variation of Fe_2_O_3_ is prominently similar to the SiO_2_ and Al_2_O_3_ trend based on the torrefied biomass and blended binder composition. Based on the XRF analyses carried out on different samples, no significant differences could be observed in the ash composition of raw coal and bio-coal briquette. By implication, the addition of torrefied biomass up to 10%, the use of blended binder (15%), and the briquetting process do not impair the ash composition, which is also a major concern for fuel briquettes^23,44^. This is obvious with the ash fusion temperatures (AFT) for the different hybridization ratios.

**Table 2:** Ash composition of the raw coal and bio-coal briquettes.

Samples	Ash compositions									
	SiO_2_ (%)	Al_2_O_3_ (%)	Fe_2_O_3_ (%)	K_2_O (%)	MgO (%)	CaO (%)	Na_2_O (%)	TiO_2_ (%)	ZnO (ppm)	CuO (ppm)
Rawcoal	58.00	19.97	8.26	2.04	2.07	2.56	0.21	1.26	0.02	0.01
**Bio-coal briquette (97:3)**										
Binder ratios										
5P-5 M	58.30	19.72	8.41	2.19	2.08	2.4	0.23	1.59	0.02	0.01
8P-7 M	58.50	20.53	8.61	2.21	1.83	2.74	0.21	1.62	0.02	0.01
10P-5 M	57.60	19.08	8.67	2.43	1.92	2.95	0.22	1.61	0.02	0.01
5P-10 M	58.00	19.83	8.29	2.24	2.00	2.64	0.21	1.59	0.02	0.01
**Bio-coal briquette (95:5)**										
Binder ratios										
5P-5 M	57.50	19.82	8.35	2.14	2.06	2.6	0.21	1.62	0.02	0.01
8P-7 M	57.36	19.86	8.32	2.14	2.01	2.81	0.22	1.61	0.02	0.01
10P-5 M	58.21	19.90	8.35	2.07	2.09	2.81	0.22	1.59	0.02	0.01
5P-10 M	58.24	20.50	8.42	2.07	2.03	2.82	0.22	1.56	0.02	0.01
**Bio-coal briquette (90:10)**										
Binder ratios										
5P-5 M	58.32	19.63	8.25	2.01	2.06	2.78	0.21	1.63	0.02	0.01
8P-7 M	58.86	19.76	8.22	2.14	2.01	2.67	0.20	1.61	0.02	0.01
10P-5 M	58.21	19.90	8.30	2.16	2.1	2.61	0.20	1.60	0.02	0.01
5P-10 M	58.24	19.80	8.32	2.09	2.03	2.66	0.21	1.54	0.02	0.01

### Ash fusion temperatures

The ash fusion temperature (AFT) of the raw coal and bio-coal briquette ashes are shown in Table 3. The AFT is a major factor that is currently used to evaluate the melting and fusibility characteristics of raw coal and bio-coal briquette ashes in its conversion. The deformation temperature (DT) ranges from 1200—1250 °C for coal and bio-coal briquette ashes, while the softening temperatures (ST) are from 1240—1290 °C. The hemispherical temperature (HT) and final fusion temperature (FT) range from 1270—1300 °C and 1300—1350 °C, respectively. The DT, HT, ST, and FT of bio-coal briquette ashes dropped slightly compared to the raw coal. Liu et al.^45^ also reported a reduction in the AFTs of Shenfu bitumite by 21 °C when the proportion of 66.7% of water hyacinth was added to the coal. However, Li and Fang^34^ reported a drop (0–10%) in the AFTs of the mixture of biomass and lignite when biomass increased in the mass ratio. This pattern was linked with the presence of low-melting-point ferrous sulphide oxide, calcium sodium sulfates, among others. It was concluded that three kinds of biomass can cause a decrease in the AFTs of lignite coal. The progression from ST to FT for the bio-coal briquettes corresponds to the melting of most of the minerals as a result of the intensive solution of refractory minerals and change in viscosity and flow properties of the melts. FT has been linked to flow changes in liquid and plastic phases. The FT of the raw coal and bio-coal briquettes do not exceed 1350 °C, whereas FT of > 1500 °C was reported for Meghalaya coal^42^. This shows that the raw coal is a lean grade type compared with Meghalaya coal, which was reported to be of higher grade (subbituminous). The AFTs of raw coal and bio-coal briquette largely depend upon the oxide ratios of the ashes. Brief descriptions of the important oxide ratios are discussed.

**Table 3:** Ash fusion temperatures of raw coal and bio-coal briquettes.

Samples	DT ( °C)	ST ( °C)	HT ( °C)	FT ( °C)
Raw coal	1250	1290	1300	1350
**Bio-coal briquette (97:3)**				
Binder ratios				
5P-5 M	1240	1260	1290	1320
8P-7 M	1230	1240	1280	1300
10P-5 M	1240	1250	1290	1330
5P-10 M	1240	1250	1270	1310
**Bio-coal briquette (95:5)**				
Binder ratios				
5P-5 M	1240	1250	1290	1340
8P-7 M	1230	1260	1280	1330
10P-5 M	1240	1240	1280	1340
5P-10 M	1230	1260	1280	1330
**Bio-coal briquette (90:10)**				
Binder ratios				
5P-5 M	1200	1250	1270	1300
8P-7 M	1210	1250	1280	1310
10P-5 M	1230	1240	1290	1320
5P-10 M	1220	1240	1270	1310

### Oxide ratios

The oxide ratios of the raw coal and bio-coal briquettes are shown in Table 4. This sub-section explains the significance of these oxides in the briquettes when in use.

**Table 4:** Oxide ratios of the raw coal and bio-coal briquettes.

Samples	Silica/alumina	Basic/acidic	Silica ratio	Slagging factor	Fouling factor
Raw coal	2.90	0.19	0.82	0.14	0.43
**Bio-coal briquette (97:3)**					
Binder ratios					
5P-5 M	2.96	0.19	0.82	0.14	0.46
8P-7 M	2.85	0.19	0.82	0.14	0.47
10P-5 M	3.02	0.21	0.81	0.15	0.55
5P-10 M	2.92	0.19	0.82	0.14	0.47
**Bio-coal briquette (95:5)**					
Binder ratios					
5P-5 M	2.90	0.19	0.82	0.14	0.46
8P-7 M	2.89	0.20	0.82	0.14	0.46
10P-5 M	2.93	0.19	0.81	0.14	0.45
5P-10 M	2.84	0.19	0.81	0.14	0.44
**Bio-coal briquette (90:10)**					
Binder ratios					
5P-5 M	2.97	0.19	0.82	0.14	0.43
8P-7 M	2.98	0.19	0.82	0.14	0.44
10P-5 M	2.93	0.19	0.82	0.14	0.46
5P-10 M	2.94	0.19	0.82	0.14	0.44

#### Silica-alumina ratios (SiO_2_/ Al_2_O_3_)

The silica-alumina ratio is an important parameter that affects the flow properties of coal ash slag. It is negatively correlated with DT, ST, and FT^39^. The silica-alumina ratio (abrasion potential), as presented in Table 4, ranges from 2.8—3.01 for raw coal and bio-coal briquette. This is a little higher than 2.01—2.86 reported for PCB coal by Mishra et al.^39^. This signifies the presence of coarse-grained non-spherical quartz, which can sustain the ash as solid until 1600°C^46,47^. However, the presence of other basic oxides in the coal and bio-coal briquette lowers the softening and melting points of the ashes to 1350 °C. The trend of inorganic matters in the coal ash is a reminiscence of the bio-coal briquette ash and this depicts that there is a little contribution from the addition of torrefied biomass, pitch and molasses to the inorganic matters in the bio-coal briquette since they are basically organic materials.

#### Silica ratio (SiO_2_/ (SiO_2_ + Fe_2_O_3_ + CaO + MgO))

These oxides ratio is applied to predict coal ash slagging performance^41,48^. Good coal must have a high silica ratio $\ge$ 0.78, which implied that it will be hard to fuse^41^. The silica ratio of both raw coal and bio-coal briquette ashes are > 0.81 as presented in Table 4. The silica ratios obtained are similar to that of Prajapara coal, which showed a positive correlation to DT and FT, and was considered as good coal^41^. Thus, the silica ratios of the present study show that the bio-coal briquette will perform well during slagging.

#### Basic/acid oxides ratio ((Fe_2_O_3_ + CaO + MgO + K_2_O + Na_2_O)/(SiO_2_ + TiO_2_ + Al_2_O_3_))

The ratio of the basic to acidic oxides is also considered as an index for slagging behaviour^49^. The basic/acidic (ratio) has been reported to have a negative correlation with FT and DT^41^. Coal with a basic/acidic (B/A) ratio $\ge$ 0.4 is grouped to be low melting ash, while coal with B/A ratio $\le$ 0.11 is considered to be very good coal for thermal and metallurgical applications. The B/A ratios of the ashes of raw coal and bio-coal briquette are in the range of 0.19—0.20, as shown in Table 4. This indicates that slag formation will be highly favoured during the use of the bio-coal briquette.

#### Slagging factors (B/A*sulphur)

The slagging factor of the ash of coal and bio-coal briquette ranges from 0.13 to 0.14, as shown in Table 4. This is lower than the slagging factor of 0.6 recommended for coal that has low slagging potential^50^. On a general basis, the slagging factor of the bio-coal briquette ash was similar to that of the raw coal, which indicates that the addition of torrefied biomass, pitch, and molasses to raw coal did not increase its slagging potential. Thus, the slagging potential of the bio-coal briquette totally depends on the mineral composition of the raw coal.

#### Fouling factor (B/A*(Na_2_O + K_2_O))

The tendency of coal to pollute the environment by smelling or generation of foul odours is usually determined by the fouling factor. The fouling factor (FF) of the bio-coal briquette ashes (0.44—0.55) were a little higher compared to that of raw coal (0.43). The FF results of the present study are similar to FF of < 0.45, which were obtained for Prajapara coal^41^ and Meghalaya coal^42^ that were reported to have a low fouling factor. The low FF indicates that the bio-coal briquette has low potential to fouling. Based on the oxides’ ratios (Table 4), the ashes of bio-coal briquette behaved similarly to that of raw coal used for its production. It is worthy to note that the ashes of bio-coal briquette does not have high tendency for clinker formation, slagging and fouling during its conversion processes such as combustion and gasification^35^.

**Figure 4:**
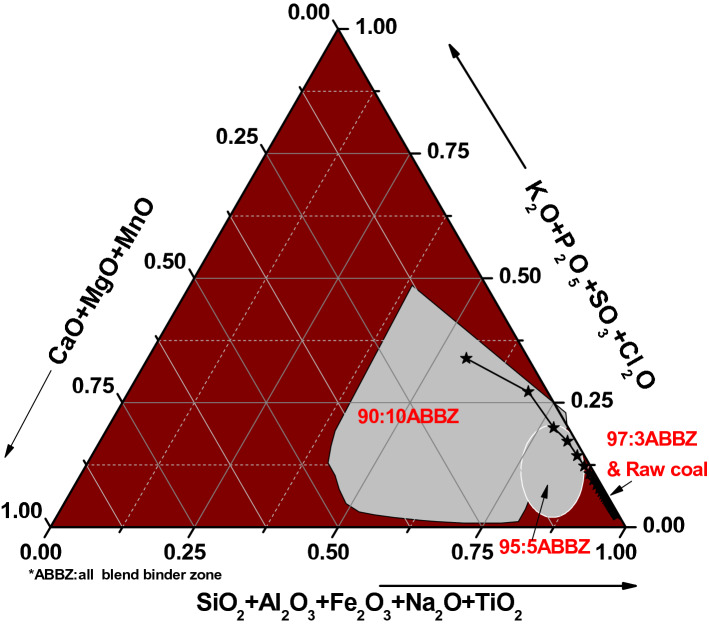
Area chemical classification of the hybrid briquette ashes.

One of the major drawbacks of using biomass as a partial replacement for coal in energy generation is the behaviour of its inorganic matters^51^. Figure 4 presents the grouping of the ashes of raw coal and bio-coal briquettes based on the ternary relationship among the detrital, authigenic, and technogenic types. Based on the grouping, the ashes of raw coal and bio-coal briquettes majorly contain detrital minerals (SiO_2_ + Al_2_O_3_ + Fe_2_O_3_ + Na_2_O + TiO_2_). This implied that the ashes contained stable, less-reactive, and high melting temperature minerals. A minimal deviation into the authigenic and technogenic groups was observed as the torrefied biomass increased to 10% of the composition of the bio-coal briquettes. These minerals are unstable, highly mobile, very reactive, and with low decomposition/melting temperature when fuels are in use. Higher content of torrefied biomass within the bio-coal briquettes may induce more of these minerals^51,52^. This low-melting- temperature composition of the ash usually leads to low combustion efficiency for fuel when in use and could increase operating cost. The tannery plot in Fig. 4 clearly showed that authigenic (CaO + MgO + MnO) and technogenic (K_2_O + P_2_O_5_ + SO_3_ + Cl_2_O) minerals increases with an increase in torrefied biomass within the bio-coal briquettes. Thus, a need to minimize the quantity of torrefied biomass in bio-coal briquettes to avoid inefficient combustion.

## Conclusion

An attempt was made to evaluate the ash composition and behaviour of bio-coal briquettes of lean grade coal and torrefied biomass produced using a blend of coal tar pitch and molasses as the binder. Quartz was conspicuously the dominant mineral phase in all the ashes of the bio-coal briquettes. The addition of torrefied biomass ($\le$ 10%) had a negligible impact on the major mineral oxides present in the ashes. The ash fusion temperature was mildly affected by the addition of torrefied biomass and blended binder as the final fusion temperature was $\approx$ 1350 °C. The mineral oxides were mostly the detrital rather than the authigenic or technogenic types. The addition of torrefied biomass ($\le$ 10%) to coal and the use of blended pitch and molasses as the binder ($\le$ 15%) have minimal influence on the ash composition of the resultant bio-coal briquette.

## Data Availability

The data will be made available upon request.
